# Urothelial protein expression in pediatric patients with ureteropelvic junction obstruction

**DOI:** 10.1007/s00467-026-07466-5

**Published:** 2026-07-10

**Authors:** Ashley R. Jackson, Sudipti Gupta, Macie Kercsmar, John W. Froehlich, Richard S. Lee, Brian Becknell, Christina B. Ching

**Affiliations:** 1 Kidney and Urinary Tract Center, The Abigail Wexner Research Institute at Nationwide Children’s Hospital, Columbus, OH USA; 2 Division of Nephrology and Hypertension, Nationwide Children’s Hospital, 700 Children’s Drive, 700 Children’s Drive, Columbus, OH 43205 USA; 3 Department of Urology, Nationwide Children’s Hospital, 700 Children’s Drive, Columbus, OH 43205 USA; 4 Department of Urology, Boston Children’s Hospital, Boston, MA USA; 5 Department of Surgery, Harvard Medical School, Boston, MA USA

**Keywords:** Ureteropelvic junction obstruction, Obstructive uropathy, Congenital urinary tract obstruction, Urothelium, Uroplakin, Keratin

## Abstract

**Background:**

Pediatric ureteropelvic junction obstruction (UPJO) is the leading cause of congenital obstructive uropathy. Experimental models have identified local urothelial remodeling in UPJO and implicated this remodeling as a protective adaptation to preserve kidney structural integrity and function. We investigated the expression of urothelial proteins in human urine and resected tissue specimens from children with UPJO in a pilot study.

**Methods:**

Children undergoing pyeloplasty for clinically significant UPJO and healthy age- and sex-matched controls were included. Bladder urine samples were evaluated by ELISA for urothelial proteins (KRT5, KRT14, KRT20, UPK2, UPK3A) and normalized to creatinine (Cr). When available, kidney urine collected at pyeloplasty was compared to bladder urine from the same subject. Urothelial proteins were localized by immunofluorescence microscopy in surgically resected kidney pelvises proximal to the obstruction.

**Results:**

Twenty-three UPJO and 14 control urines were evaluated. KRT14/Cr, KRT20/Cr, and UPK2/Cr levels were significantly higher in UPJO urine compared to controls (*p* < 0.05; Mann–Whitney *U* test). Levels of urothelial proteins were as high or higher in urine obtained from the obstructed kidney when compared to paired bladder urine. Immunofluorescence microscopy of obstructed urothelium identified basal KRT14+ cells, pan-urothelial UPK+ cells, and superficial KRT20 + cells, with nuclear localization of two transcription factors implicated in obstructive urothelial remodeling, GRHL3 and PPARγ.

**Conclusions:**

We provide evidence of increased urothelial proteins in urine from children with UPJO in this pilot study, along with evidence of their local expression by obstructed urothelium.

**Graphical abstract:**

**Figure:**
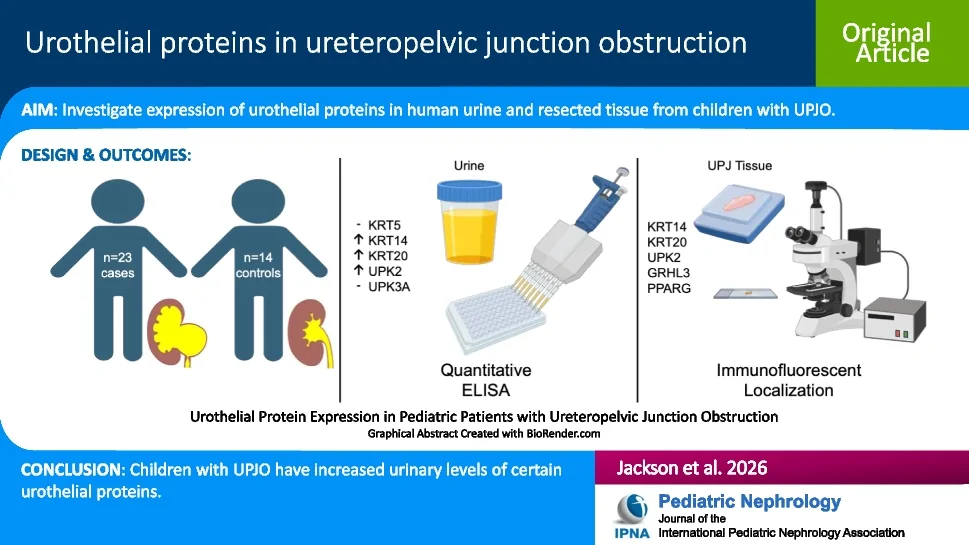
A higher resolution version of the Graphical abstract is available as Supplementary information.

**Supplementary Information:**

The online version contains supplementary material available at https://doi.org/10.1007/s00467-026-07466-5.

## Introduction

Congenital ureteropelvic junction obstruction (UPJO) can lead to kidney parenchymal injury and decline in kidney function. Surgical intervention through pyeloplasty in cases of significant UPJO can stabilize and even improve kidney function, but differentiating significant UPJO from non-obstructive hydronephrosis in the pediatric population can be particularly challenging [1]. While symptoms can point toward clinically relevant obstruction in adults, these are not typical triggers to identify potentially injurious UPJO in young children. As a result, pediatric providers generally rely on imaging, such as ultrasound and nuclear scintigraphy, that might suggest significant UPJO. Unfortunately, hydronephrosis alone is unable to identify pathologic obstruction. In addition, pediatric imaging can be influenced by inherent properties of abnormal kidney parenchymal development or intrinsic properties of the pediatric kidney pelvis [2], further clouding the picture. This scenario has galvanized the investigation of urinary proteins and peptides to serve as noninvasive biomarkers of kidney deterioration and thereby assist in the identification of significant UPJO [1, 3–5].

The urothelial lining of the kidney pelvicalyceal system and papilla is significantly impacted by UPJO. Histology studies of resected UPJ specimens at pyeloplasty have identified increased urothelial thickening and hyperplasia as characteristic changes within the obstructed segment [6, 7], along with loss of apical Uroplakin (UPK) III expression and increases in intercellular spacing, suggestive of urothelial injury [7]. Experimental models of urinary tract obstruction have further substantiated this concept. Unilateral ureteral obstruction (UUO) results in urothelial desquamation, disrupted tight junctions, urine leak, loss of apical UPK, and enlarged intercellular spacing—suggestive of defects in urothelial barrier function [8, 9]. Subsequent proliferation of KRT5+ and KRT14+ basal cells leads to thickening of the UPJ urothelium as well as the intrarenal urothelium that covers the kidney papilla [8, 10]. In addition to this urothelial expansion, apical cells within the intrarenal urothelium hypertrophy and upregulate expression of UPK proteins, which assemble an apical urothelial plaque, in a manner dependent on transcription factors that promote urothelial differentiation, including Peroxisome Proliferator-Activated Receptor γ (PPARγ) and Grainyhead-like 3 (GRHL3) [8, 11–13]. Ultimately, these local changes in urothelial structure are functionally beneficial, as genetic depletion of urothelial plaque, Upk+ cells, or PPARγ accelerates kidney parenchymal loss [11, 12]. Conversely, targeted expression of a constitutively active PPARγ-VP16 protein in Upk+ cells drives a transcriptional program of urothelial differentiation that attenuates parenchymal loss during UUO [12, 14]. These studies substantiate that urothelial remodeling is an adaptive process that serves to protect the kidney from obstructive injury [11, 12].

Along these lines, urothelial proteins and peptides may serve as indicators of clinically significant UPJO. Consistent with this hypothesis, certain peptides and proteins that are elevated in patients with UPJO compared to healthy controls originate at least in part from urothelium, including HIP/PAP, CA19-9, and NGAL [5, 15, 16], but also may derive from kidney tubules. In the present study, we tested the hypothesis that urothelial-specific proteins are elevated in urine samples from patients with UPJO at the time of pyeloplasty in comparison to healthy controls. In addition, we utilized immunofluorescence microscopy in resected tissue to map the distribution of these proteins in obstructed urothelium.

## Methods

### Urine samples of pediatric patients undergoing surgery for UPJO

All patients were recruited at Nationwide Children’s Hospital in Columbus Ohio after Institutional Review Board approval (IRB14-00087). Bladder urine samples were collected with informed consent/assent from pediatric patients (≤ 21 years old) at the time of pyeloplasty for presumed UPJO (“UPJO cohort”) as previously described [5, 17]. All subjects underwent diuretic scintigraphy and renal ultrasound, and all had Society of Fetal Urology (SFU) grade 2 or higher unilateral hydronephrosis (Table 1). Briefly, patients were considered to have obstructive pathology from their ureteropelvic junction that required surgical intervention as deemed appropriate by their treating pediatric urologists. Indications for surgery included hydronephrosis with decreased split kidney function on nuclear scintigraphy, hydronephrosis with pain, worsening hydronephrosis without hydroureteronephrosis on ultrasound, and any decline in ipsilateral relative kidney function on nuclear scintigraphy with hydronephrosis. Bladder urine was collected by urethral catheter immediately prior to surgical incision and before surgical decompression. When possible, urine samples were also collected via direct aspiration from the affected kidney during surgery. Patients were excluded if they demonstrated signs of UTI on urinalysis (i.e., positive nitrites or more than trace leukocyte esterase positivity) and/or UTI symptomatology at time of collection.

**Table 1:** Demographics of patients in the experimental group undergoing surgery for UPJO and those of healthy controls

	UPJO (*n* = 23)	Controls (*n* = 14)	*p* value
Median age years (IQR)	3.4 (1.7, 7.4)	1.9 (0.5, 5.5)	0.2343
Male (%)	14 (61)	10 (71)	0.5141
SFU grade 2 3 4	2 6 15		
Median ipsilateral T1/2 drainage on renal scan prior to surgery (IQR)	36 (21.6, 81.6)		
Median ipsilateral split renal function % (IQR)	52 (45, 56)		
Crossing vessel found at surgery	5		

### Control urine samples from healthy pediatric patients

Control urine samples were collected from children treated in the Urology Department for non-urinary tract pathology (e.g., orchiopexy for cryptorchidism) or at the time of well child visits to our hospital’s primary care clinic. “Control” samples were chosen to closely match age and sex of UPJO cases. These samples consisted of bladder urine collected via free void, application of a urine bag, or catheterization. Controls were excluded if they had a history of abnormal prenatal or postnatal kidney imaging, prematurity, history of UTI or urinary tract surgery, renal dysfunction, chronic kidney disease, or evidence of UTI on urinalysis at the time of collection. A review of the electronic medical record was performed to corroborate this information. Controls did not undergo renal imaging to confirm normal anatomy and absence of obstruction.

### Urine sample processing

Urinalysis of all urine samples was performed by dipstick (Siemens Healthcare Diagnostics, Inc., Tarrytown, NY) immediately after collection, after which a protease inhibitor (Assay Assure, Sierra Molecular, Incline Village, NV) was added. Samples were then aliquoted and stored at −80 °C until analysis [5]. Samples were centrifuged at 750 g for 8 min at 4 °C, followed by 3000 rpm for 5 min to remove cellular debris and restricted to a single freeze/thaw cycle before analysis.

### Measurement of urinary urothelial proteins

We performed enzyme-linked immunosorbent assays (ELISA) on urine samples of the UPJO cohort and controls using commercially available kits for KRT5 (Cloud-Clone, Katy TX), KRT14 (Cloud-Clone), KRT20 (Cloud-Clone), UPK2 (LSBio, Seattle, WA), and UPK3A (LSBio), per the manufacturers’ instructions. ELISAs for KRT5, KRT20, and UPK2 were run undiluted. UPK3A was run at a 1:3 dilution, and KRT14 was run at 1:10. For matched patient “Bladder” and “Kidney” samples, UPK2, UPK3A, KRT14, and KRT5 were run undiluted, while KRT20 was run at 1:10. ELISAs were performed in duplicate on cell-free supernatants. Urothelial protein levels were normalized to urine creatinine (Cr), measured using colorimetric assay (Oxford Biomedical Research, Rochester Hills, MI), and expressed as a ratio to UCr (ng protein/mg Cr). The limits of detection for each analyte were KRT5 (15.6–1,000 pg/mL), KRT14 (0.156–10 ng/mL), KRT20 (0.156–10 ng/mL), UPK2 (0.157–10 ng/mL), UPK3A (0.313–20 ng/mL), and Cr (1–10 mg/dL).

### Immunofluorescence microscopy

Kidney pelvis tissue just above the UPJ (obstructed urothelium) and beyond that required for clinical pathologic evaluation was collected with informed consent/assent at pyeloplasty. The kidney pelvis from a child who underwent pre-emptive bilateral nephrectomy for medication refractory hypertension related to kidney failure secondary to glomerulosclerosis of unknown etiology, who lacked evidence of UPJO, was included for comparison. Tissue samples from pyeloplasty were fixed in 10% formalin, embedded in paraffin, and serially sectioned at 4 μm. Following deparaffinization and rehydration, antigen retrieval was achieved by submerging slides in sodium citrate buffer in a pressure cooker. Nonspecific binding was blocked using SuperBlock (ScyTek Laboratories, Logan, UT). Antibodies to GRHL3 (1:800 #ab221058, Abcam), KRT14 (1:800 #905304, Biolegend), KRT20 (1:200 #18–2200, Invitrogen), PPARG (1:400, #2435S, Cell Signaling Technology), UPK2 (1:300 #21149-1-AP, Proteintech), and species-specific Cy3 and Alexa Fluor 488 secondary antibodies (1:300, Jackson ImmunoResearch Laboratories, West Grove, PA) were used. Nuclei were stained with Hoechst 33342 (1 µg/mL, #PI62249, Thermo Fisher Scientific). Coverslips were mounted using ProLong Gold Antifade Mountant (#P36930, Thermo Fisher Scientific), and images were captured using a Nikon Ti2-E microscope (Nikon Instruments Inc., Melville, NY), equipped with a Hamamatsu ORCA-Fusion camera (Hamamatsu Japan).

### Statistical analysis

The D’Agostino–Pearson omnibus normality test determined if data were parametric between groups. For evaluation of kidney and bladder samples, the Shapiro–Wilk test was used to evaluate normality due to the small sample size. Between group comparisons were found to be nonparametric, and thus, the Mann–Whitney *U* test was performed. For comparison of kidney versus bladder urine samples from the same subject, a paired *t*-test analysis was performed. The chi-square test was used for categorical data. Correlations between urothelial protein levels were evaluated using Spearman’s rank test. A *p* value < 0.05 was considered significant. GraphPad Prism 10.6.0 (GraphPad Software Inc., La Jolla, CA) was used for these analyses.

## Results

### Patient demographics

We recruited 23 assenting/consenting UPJO patients undergoing pyeloplasty as part of this pilot study. Median age was 3.4 years (IQR 1.7, 7.4) and 14 (61%) were male. There was no significant difference in age or gender between those undergoing UPJO and controls (Table 1). Upon review of intraoperative findings in the UPJO cohort, five patients (21.7%) had a crossing vessel while the other 18 patients had an isolated intrinsic UPJ abnormality.

### Urothelial proteins in the urine were higher in children with UPJO than controls

**Fig. 1:**
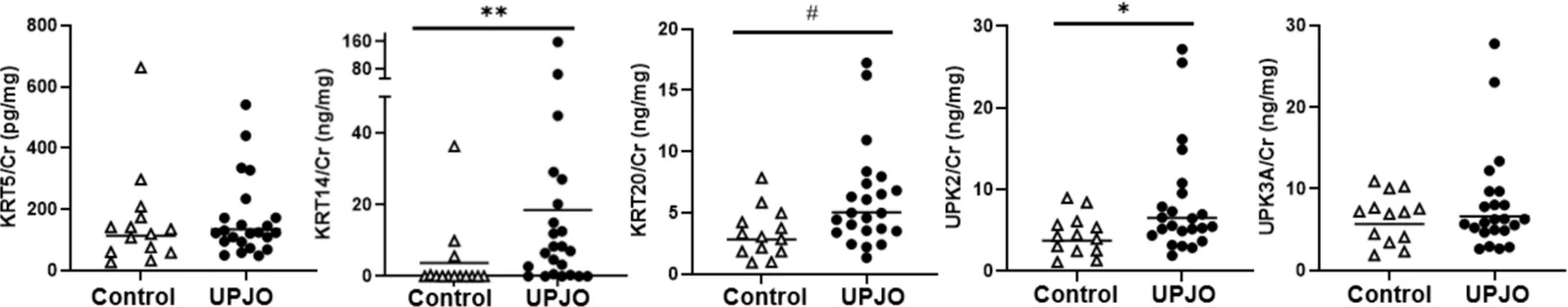
Urinary urothelial proteins in subjects undergoing pyeloplasty for UPJO and age- and sex-matched controls (*p = 0.0471; **p = 0.0047; #*p* = 0.0133; Mann–Whitney *U* test). The horizontal line indicates the geometric mean (KRT5/Cr, KRT20/Cr, UPK2/Cr, and UPK3A/Cr) or mean (KRT14/Cr)

**Fig. 2:**
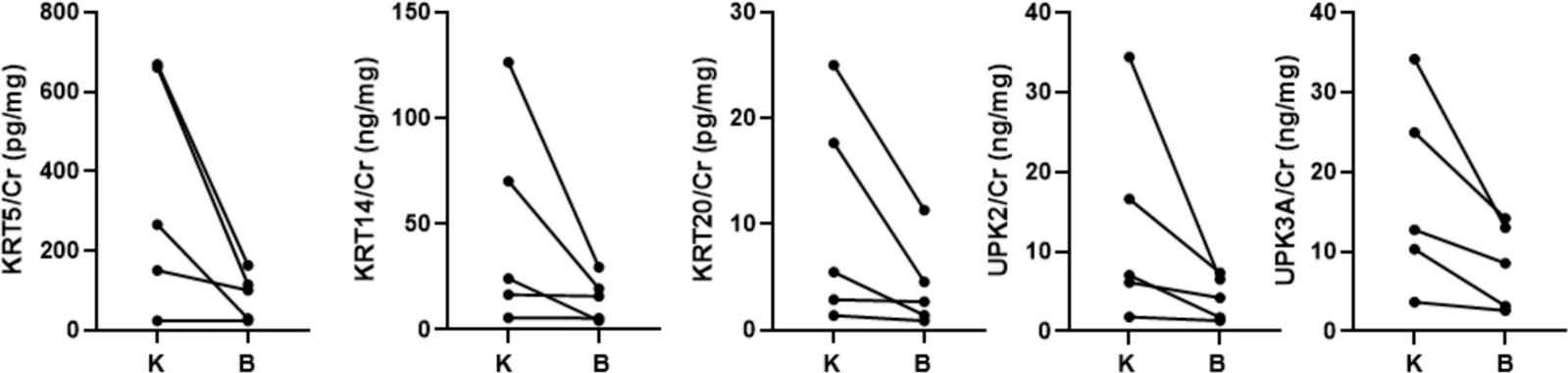
Paired kidney (K) vs. bladder (B) urines from the same subject with UPJO obtained at the time of surgery (*n* = 5). There were no significant differences between kidney and bladder urine levels of any analyte (paired *t-*test; KRT5/Cr *p* = 0.0764; KRT14/Cr *p* = 0.1387; KRT20/Cr *p* = 0.1008; UPK2/Cr *p* = 0.1447; UPK3A/Cr *p* = 0.5247)

There was significantly higher KRT20, UPK2, and KRT14 expression in the urine of patients with UPJO compared to controls (Fig. 1), but KRT5 and UPK3A levels were comparable. Levels of urothelial proteins were as high or higher in urine obtained from the obstructed kidney when compared to paired bladder urine (Fig. 2). Levels of KRT5, KRT20, UPK3A, and UPK2 correlated with one another by Spearman’s rank test. There was no significant association of urothelial protein concentration and SFU hydronephrosis grade and ipsilateral differential renal function of the UPJO kidney on nuclear scintigraphy. There were no significant differences in KRT20, UPK2, or KRT14 levels between the patients with crossing vessel versus intrinsic abnormality as a cause of their UPJO.

### Local expression of urothelial proteins in obstructed urothelium

**Fig. 3:**
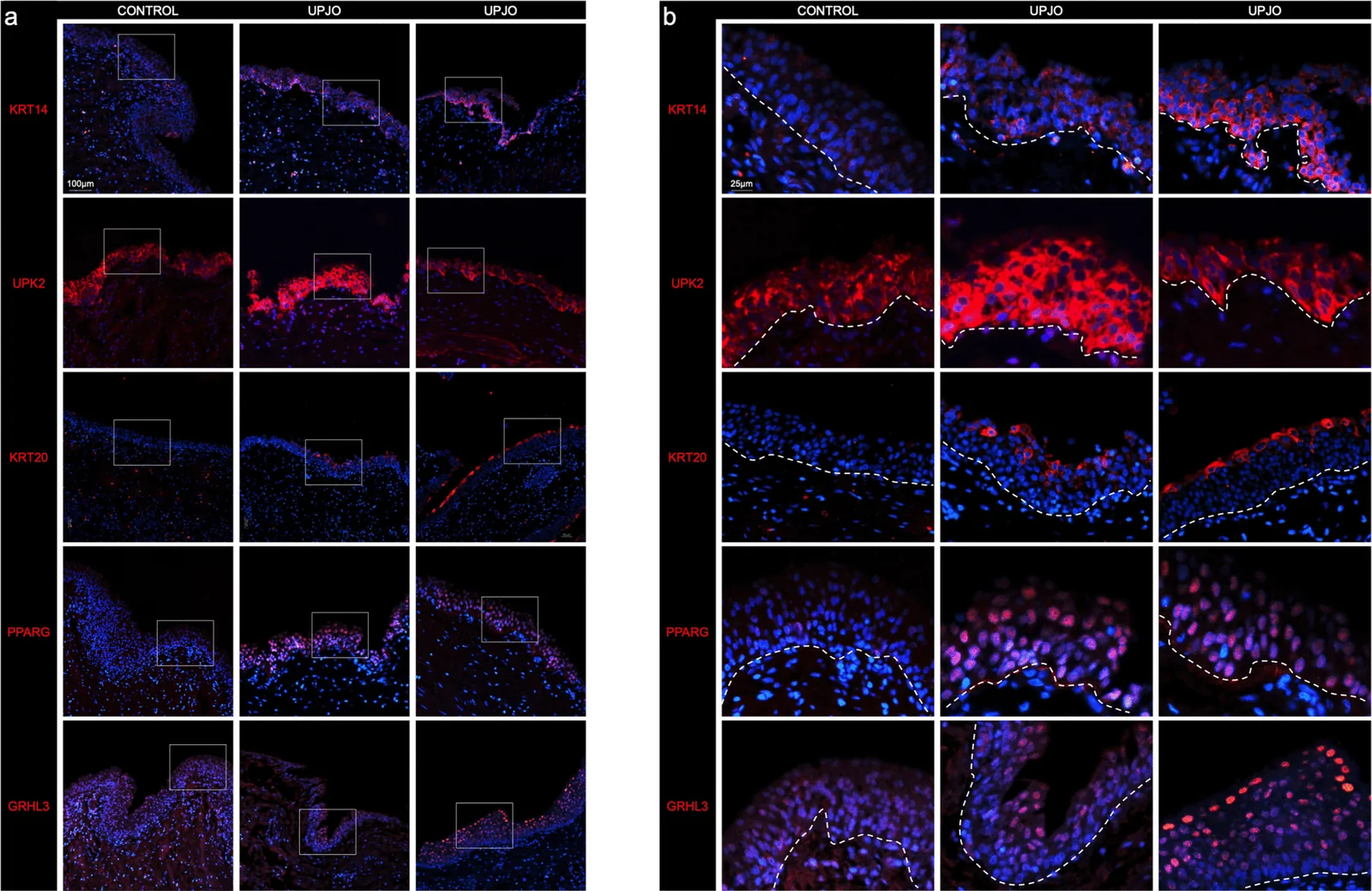
The distribution of proteins in obstructed urothelium from two subjects undergoing pyeloplasty for UPJO was evaluated by immunofluorescence microscopy. Pelvic urothelium from a subject undergoing nephrectomy for medication refractory hypertension was available for comparison (control). UPJO images are representative of findings in five subjects undergoing pyeloplasty. **a** Representative micrographs show KRT14, UPK2, KRT20, PPARγ, and GRHL3 immunolabeling with Hoechst 33342 nuclear counterstain in unobstructed and UPJO urothelium. **b** Higher resolution micrographs correspond to insets in (**a**). White dashed lines: urothelial basement membrane. Original magnification: ×30 (×20 objective with ×1.5 optical zoom). Staining patterns are consistent with published protein localization (cytoplasmic for KRT14 and KRT20, membrane-associated and cytoplasmic for UPK2, and nuclear for PPARγ and GRHL3) [8, 12, 18]

Urothelial proteins were chosen for immunofluorescence microscopy based on their increased concentration in obstructed urine. Given their roles in urothelial remodeling during obstructive uropathy, we also evaluated the distribution of PPARγ and GRHL3 [12]. Obstructed urothelium from the pelvis of five subjects undergoing pyeloplasty was available for review. The kidney pelvis from one subject who underwent pre-emptive nephrectomy for management of hypertensive complications of kidney failure was available for comparison. In specimens from patients with UPJO, basal and intermediate urothelial cells expressed KRT14, while UPK2 showed pan-urothelial expression and KRT20 was restricted to superficial cells. Nuclear PPARγ was distributed throughout the urothelium, and nuclear GRHL3 was enriched in apical cell layers. In the unobstructed UPJ, UPK2 and GRHL3 displayed a similar distribution, whereas expression of KRT14, KRT20, and PPARγ was less evident (Fig. 3).

## Discussion

Management of pediatric UPJO is complicated by uncertainty surrounding clinically significant urinary tract obstruction. While pyeloplasty offers the promise of definitive surgical repair, the high rate of spontaneous resolution of radiologic lesions suggestive of UPJO calls the need for surgery into question and contributes to the conundrum facing pediatric urologists. To meet this challenge, noninvasive biomarkers of parenchymal injury may serve to resolve the ambiguity around diagnosis of clinically significant UPJO. Here, we address the impact of UPJO on the urothelium by investigating urothelial structural proteins from the obstructed urothelium and measuring urine levels of these proteins at the time of pyeloplasty in a pilot study. Our results demonstrate that a subset of urothelial proteins consisting of KRT14, KRT20, and UPK2 is significantly elevated in UPJO urine compared to age- and sex-matched healthy controls. In addition, we detected local expression of these proteins in obstructed urothelium from subjects with UPJO at the time of pyeloplasty, accompanied by nuclear localization of transcription factors implicated in urothelial differentiation and remodeling. Altogether, our findings implicate obstructed urothelium as a source of proteins that accumulate in the urine of pediatric patients with UPJO.

Previous studies have identified increased expression of urinary proteins in patients with UPJO that derive at least in part from the urothelium, such as CA19-9 and HIP/PAP [5, 15]. Here, we extend these findings by demonstrating that a subset of urothelial proteins—KRT14, KRT20, and UPK2—is increased in urine obtained from the bladder at the time of pyeloplasty compared to unaffected controls. Levels of these proteins in urine aspirated directly from the affected kidney were as high or higher than those obtained concurrently from the bladder, consistent with the hypothesis that their elevation in bladder urine is reflective of UPJO. Future work in larger patient cohorts will clarify whether sampling location impacts urine protein concentrations. In addition, prospective cohorts of patients with both obstructive and non-obstructive hydronephrosis will establish the specificity of these proteins’ association with UPJO.

The distribution of urothelial proteins of the urothelium above the UPJ of patients undergoing pyeloplasty supports experimental observations in laboratory mice, that urinary tract obstruction triggers a program of remodeling in the obstructed urothelium lining the kidney pelvis as well as the intrarenal urothelium [8, 10]. This remodeling is characterized by an initial loss of UPK expression, followed by expansion of K5+ and K14+ basal cells and increased production of UPK3A and UPK2 by apical cells, which secrete a protective urothelial plaque. As in the bladder, the urothelial plaque likely serves to both foster integrity of the blood:urine permeability barrier within the obstructed kidney and to promote compliance such that the extra volume of urine retained in the obstructed kidney is maintained at low pressures [19, 20]. These changes reflect physiologic adaptations within the urothelium of the obstructed kidney, as experimental depletion of the urothelial plaque, Upk2+ cells, or PPARγ—a master regulator of urothelial differentiation—accelerates kidney parenchymal loss and kidney functional impairment [11, 12]. We noted nuclear expression of both PPARγ and GRHL3 in obstructed UPJ, consistent with their roles in urothelial remodeling and differentiation in preclinical models [12, 13, 21, 22].

This was a single-center retrospective study with a small sample size, and the results should be validated and extended in a multicenter, prospective format. The lack of longitudinal follow-up in this pilot study makes it impossible to determine whether these urothelial proteins increase over time as obstruction progresses or decrease after surgical relief. Moreover, an ideal biomarker of UPJO will identify patients with obstructive kidney injury at the time of initial radiologic testing, i.e., nuclear scintigraphy and kidney ultrasound, and distinguish UPJO from non-obstructive causes of hydronephrosis, such as vesicoureteral reflux. Thus, future prospective evaluation of these urothelial proteins should include control groups with non-obstructive hydronephrosis and those with UPJO who experience spontaneous resolution and do not develop surgical indications. Lack of confirmatory imaging to rule out a renal abnormality in control subjects and variability in how urine was collected (free void vs. catheterization) in the control group compared to urine being collected by catheterization exclusively in the obstructed group are additional study limitations. Furthermore, additional UPJO and healthy, non-obstructed tissue specimens are required to test the generalizability of our observations by immunofluorescence microscopy and relate urothelial protein distribution to local histopathological features such as inflammation and fibrosis. In conclusion, pediatric patients with UPJO manifest increased urinary levels of certain urothelial proteins relative to unaffected controls, and further studies are warranted to test the clinical significance of our findings.

## Supplementary Information

Below is the link to the electronic supplementary material.

Graphical abstract (PPTX 532 KB)

## Data Availability

All data regarding urothelial protein levels and localization are available upon request from the authors.
